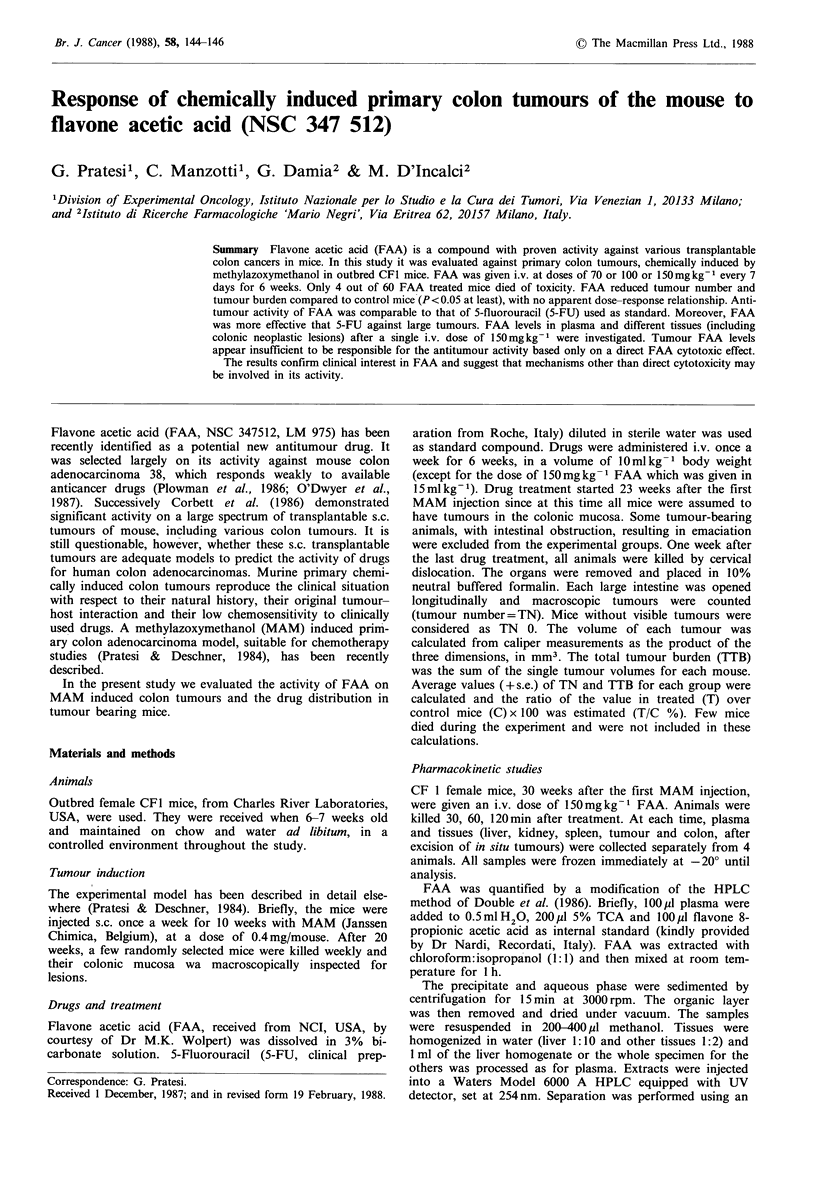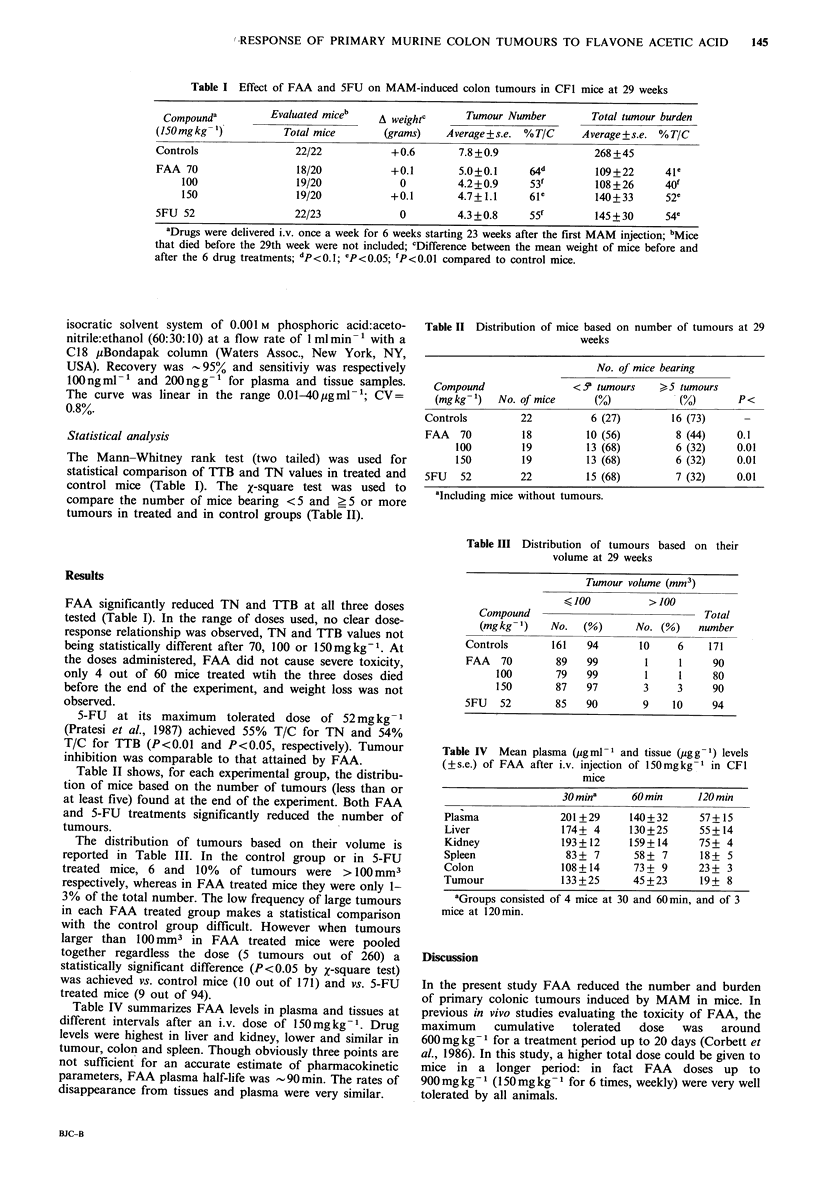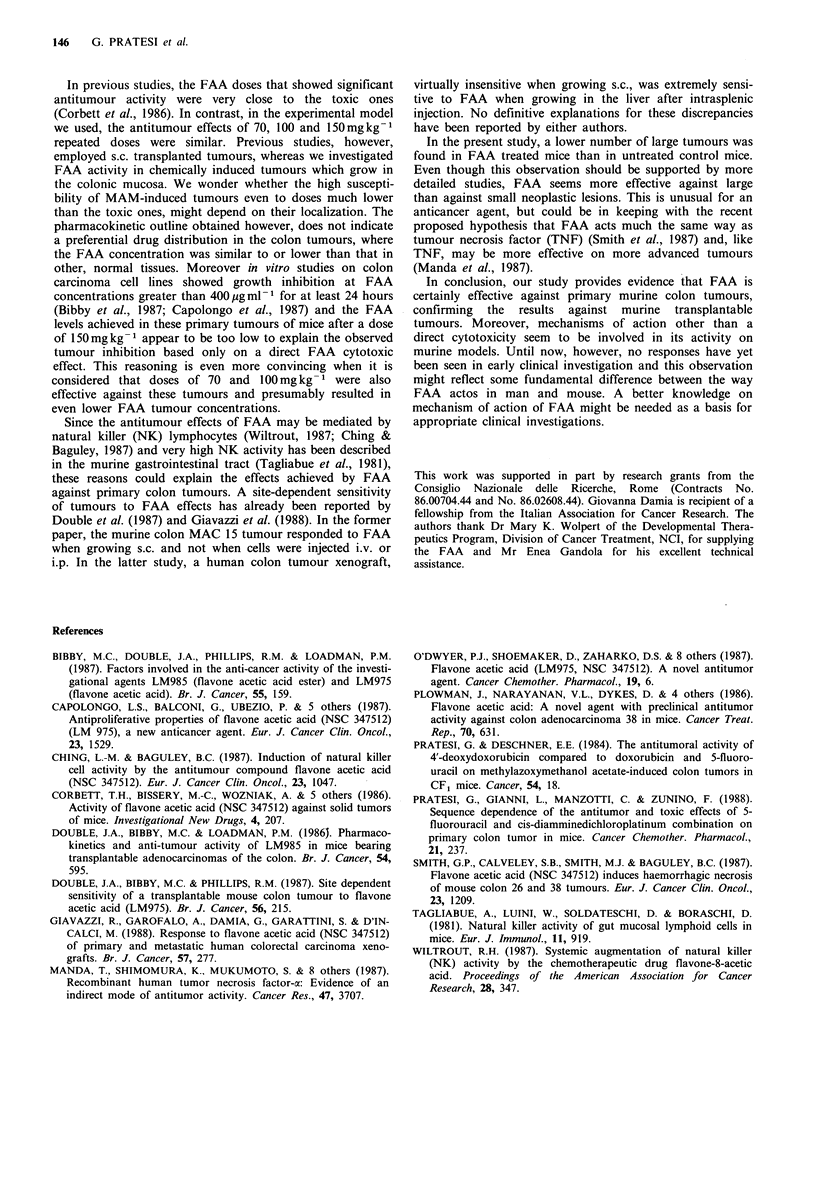# Response of chemically induced primary colon tumours of the mouse to flavone acetic acid (NSC 347 512).

**DOI:** 10.1038/bjc.1988.181

**Published:** 1988-08

**Authors:** G. Pratesi, C. Manzotti, G. Damia, M. D'Incalci

**Affiliations:** Division of Experimental Oncology, Istituto Nazionale per lo Studio e la Cura dei Tumori, Milano, Italy.

## Abstract

Flavone acetic acid (FAA) is a compound with proven activity against various transplantable colon cancers in mice. In this study it was evaluated against primary colon tumours, chemically induced by methylazoxymethanol in outbred CF1 mice. FAA was given i.v. at doses of 70 or 100 or 150 mg kg-1 every 7 days for 6 weeks. Only 4 out of 60 FAA treated mice died of toxicity. FAA reduced tumour number and tumour burden compared to control mice (P less than 0.05 at least), with no apparent dose-response relationship. Antitumour activity of FAA was comparable to that of 5-fluorouracil (5-FU) used as standard. Moreover, FAA was more effective that 5-FU against large tumours. FAA levels in plasma and different tissues (including colonic neoplastic lesions) after a single i.v. dose of 150 mg kg-1 were investigated. Tumour FAA levels appear insufficient to be responsible for the antitumour activity based only on a direct FAA cytotoxic effect. The results confirm clinical interest in FAA and suggest that mechanisms other than direct cytotoxicity may be involved in its activity.


Br. J. Cancer (1988), 58, 144–146 © The Macmillan Press Ltd., 1988

### Response of chemically induced primary colon tumours of the mouse to flavone acetic acid (NSC 347 512)

G. Pratesi[1], C. Manzotti[1], G. Damia[2] & M. D'Incalci[2]

[1]*Division of Experimental Oncology, Istituto Nazionale per lo Studio e la Cura dei Tumori, Via Venezian 1, 20133 Milano; and* [2]*Istituto di Ricerche Farmacologiche 'Mario Negri', Via Eritrea 62, 20157 Milano, Italy.*

**Summary** Flavone acetic acid (FAA) is a compound with proven activity against various transplantable colon cancers in mice. In this study it was evaluated against primary colon tumours, chemically induced by methylazoxymethanol in outbred CF1 mice. FAA was given i.v. at doses of 70 or 100 or 150 mg kg$^{-1}$ every 7 days for 6 weeks. Only 4 out of 60 FAA treated mice died of toxicity. FAA reduced tumour number and tumour burden compared to control mice ($P<0.05$ at least), with no apparent dose–response relationship. Antitumour activity of FAA was comparable to that of 5-fluorouracil (5-FU) used as standard. Moreover, FAA was more effective that 5-FU against large tumours. FAA levels in plasma and different tissues (including colonic neoplastic lesions) after a single i.v. dose of 150 mg kg$^{-1}$ were investigated. Tumour FAA levels appear insufficient to be responsible for the antitumour activity based only on a direct FAA cytotoxic effect.

The results confirm clinical interest in FAA and suggest that mechanisms other than direct cytotoxicity may be involved in its activity.

Flavone acetic acid (FAA, NSC 347512, LM 975) has been recently identified as a potential new antitumour drug. It was selected largely on its activity against mouse colon adenocarcinoma 38, which responds weakly to available anticancer drugs (Plowman *et al.*, 1986; O'Dwyer *et al.*, 1987). Successively Corbett *et al.* (1986) demonstrated significant activity on a large spectrum of transplantable s.c. tumours of mouse, including various colon tumours. It is still questionable, however, whether these s.c. transplantable tumours are adequate models to predict the activity of drugs for human colon adenocarcinomas. Murine primary chemically induced colon tumours reproduce the clinical situation with respect to their natural history, their original tumour–host interaction and their low chemosensitivity to clinically used drugs. A methylazoxymethanol (MAM) induced primary colon adenocarcinoma model, suitable for chemotherapy studies (Pratesi & Deschner, 1984), has been recently described.

In the present study we evaluated the activity of FAA on MAM induced colon tumours and the drug distribution in tumour bearing mice.

### Materials and methods

### *Animals*

Outbred female CF1 mice, from Charles River Laboratories, USA, were used. They were received when 6–7 weeks old and maintained on chow and water *ad libitum*, in a controlled environment throughout the study.

### *Tumour induction*

The experimental model has been described in detail elsewhere (Pratesi & Deschner, 1984). Briefly, the mice were injected s.c. once a week for 10 weeks with MAM (Janssen Chimica, Belgium), at a dose of 0.4 mg/mouse. After 20 weeks, a few randomly selected mice were killed weekly and their colonic mucosa wa macroscopically inspected for lesions.

### *Drugs and treatment*

Flavone acetic acid (FAA, received from NCI, USA, by courtesy of Dr M.K. Wolpert) was dissolved in 3% bicarbonate solution. 5-Fluorouracil (5-FU, clinical preparation from Roche, Italy) diluted in sterile water was used as standard compound. Drugs were administered i.v. once a week for 6 weeks, in a volume of 10 ml kg$^{-1}$ body weight (except for the dose of 150 mg kg$^{-1}$ FAA which was given in 15 ml kg$^{-1}$). Drug treatment started 23 weeks after the first MAM injection since at this time all mice were assumed to have tumours in the colonic mucosa. Some tumour-bearing animals, with intestinal obstruction, resulting in emaciation were excluded from the experimental groups. One week after the last drug treatment, all animals were killed by cervical dislocation. The organs were removed and placed in 10% neutral buffered formalin. Each large intestine was opened longitudinally and macroscopic tumours were counted (tumour number = TN). Mice without visible tumours were considered as TN 0. The volume of each tumour was calculated from caliper measurements as the product of the three dimensions, in mm$^3$. The total tumour burden (TTB) was the sum of the single tumour volumes for each mouse. Average values (+s.e.) of TN and TTB for each group were calculated and the ratio of the value in treated (T) over control mice (C) $\times$ 100 was estimated (T/C %). Few mice died during the experiment and were not included in these calculations.

### *Pharmacokinetic studies*

CF 1 female mice, 30 weeks after the first MAM injection, were given an i.v. dose of 150 mg kg$^{-1}$ FAA. Animals were killed 30, 60, 120 min after treatment. At each time, plasma and tissues (liver, kidney, spleen, tumour and colon, after excision of *in situ* tumours) were collected separately from 4 animals. All samples were frozen immediately at $-20°$ until analysis.

FAA was quantified by a modification of the HPLC method of Double *et al.* (1986). Briefly, 100 $\mu$l plasma were added to 0.5 ml $H_2O$, 200 $\mu$l 5% TCA and 100 $\mu$l flavone 8-propionic acetic acid as internal standard (kindly provided by Dr Nardi, Recordati, Italy). FAA was extracted with chloroform:isopropanol (1:1) and then mixed at room temperature for 1 h.

The precipitate and aqueous phase were sedimented by centrifugation for 15 min at 3000 rpm. The organic layer was then removed and dried under vacuum. The samples were resuspended in 200–400 $\mu$l methanol. Tissues were homogenized in water (liver 1:10 and other tissues 1:2) and 1 ml of the liver homogenate or the whole specimen for the others was processed as for plasma. Extracts were injected into a Waters Model 6000 A HPLC equipped with UV detector, set at 254 nm. Separation was performed using an

Correspondence: G. Pratesi.
Received 1 December, 1987; and in revised form 19 February, 1988.

**Table I** Effect of FAA and 5FU on MAM-induced colon tumours in CF1 mice at 29 weeks

| *Compound*[a] (*150 mg kg*$^{-1}$) | *Evaluated mice*[b] / *Total mice* | Δ *weight*[c] (*grams*) | *Tumour Number* *Average*±*s.e.* | *%T/C* | *Total tumour burden* *Average*±*s.e.* | *%T/C* |
|---|---|---|---|---|---|---|
| Controls | 22/22 | +0.6 | 7.8±0.9 | | 268±45 | |
| FAA 70 | 18/20 | +0.1 | 5.0±0.1 | 64[d] | 109±22 | 41[e] |
| 100 | 19/20 | 0 | 4.2±0.9 | 53[f] | 108±26 | 40[f] |
| 150 | 19/20 | +0.1 | 4.7±1.1 | 61[e] | 140±33 | 52[e] |
| 5FU 52 | 22/23 | 0 | 4.3±0.8 | 55[f] | 145±30 | 54[e] |

[a]Drugs were delivered i.v. once a week for 6 weeks starting 23 weeks after the first MAM injection; [b]Mice that died before the 29th week were not included; [c]Difference between the mean weight of mice before and after the 6 drug treatments; [d]$P<0.1$; [e]$P<0.05$; [f]$P<0.01$ compared to control mice.

isocratic solvent system of 0.001 M phosphoric acid:acetonitrile:ethanol (60:30:10) at a flow rate of 1 ml min$^{-1}$ with a C18 μBondapak column (Waters Assoc., New York, NY, USA). Recovery was ~95% and sensitiviy was respectively 100 ng ml$^{-1}$ and 200 ng g$^{-1}$ for plasma and tissue samples. The curve was linear in the range 0.01–40 μg ml$^{-1}$; CV= 0.8%.

*Statistical analysis*

The Mann–Whitney rank test (two tailed) was used for statistical comparison of TTB and TN values in treated and control mice (Table I). The χ-square test was used to compare the number of mice bearing <5 and ≧5 or more tumours in treated and in control groups (Table II).

**Table II** Distribution of mice based on number of tumours at 29 weeks

| *Compound* (*mg kg*$^{-1}$) | *No. of mice* | *No. of mice bearing* <5[a] *tumours* (%) | ≥5 *tumours* (%) | *P*< |
|---|---|---|---|---|
| Controls | 22 | 6 (27) | 16 (73) | – |
| FAA 70 | 18 | 10 (56) | 8 (44) | 0.1 |
| 100 | 19 | 13 (68) | 6 (32) | 0.01 |
| 150 | 19 | 13 (68) | 6 (32) | 0.01 |
| 5FU 52 | 22 | 15 (68) | 7 (32) | 0.01 |

[a]Including mice without tumours.

**Table III** Distribution of tumours based on their volume at 29 weeks

| *Compound* (*mg kg*$^{-1}$) | *Tumour volume* (*mm*$^3$) ≤*100* *No.* | (%) | >*100* *No.* | (%) | *Total number* |
|---|---|---|---|---|---|
| Controls | 161 | 94 | 10 | 6 | 171 |
| FAA 70 | 89 | 99 | 1 | 1 | 90 |
| 100 | 79 | 99 | 1 | 1 | 80 |
| 150 | 87 | 97 | 3 | 3 | 90 |
| 5FU 52 | 85 | 90 | 9 | 10 | 94 |

### Results

FAA significantly reduced TN and TTB at all three doses tested (Table I). In the range of doses used, no clear dose-response relationship was observed, TN and TTB values not being statistically different after 70, 100 or 150 mg kg$^{-1}$. At the doses administered, FAA did not cause severe toxicity, only 4 out of 60 mice treated wtih the three doses died before the end of the experiment, and weight loss was not observed.

5-FU at its maximum tolerated dose of 52 mg kg$^{-1}$ (Pratesi *et al.*, 1987) achieved 55% T/C for TN and 54% T/C for TTB ($P<0.01$ and $P<0.05$, respectively). Tumour inhibition was comparable to that attained by FAA.

Table II shows, for each experimental group, the distribution of mice based on the number of tumours (less than or at least five) found at the end of the experiment. Both FAA and 5-FU treatments significantly reduced the number of tumours.

The distribution of tumours based on their volume is reported in Table III. In the control group or in 5-FU treated mice, 6 and 10% of tumours were >100 mm$^3$ respectively, whereas in FAA treated mice they were only 1–3% of the total number. The low frequency of large tumours in each FAA treated group makes a statistical comparison with the control group difficult. However when tumours larger than 100 mm$^3$ in FAA treated mice were pooled together regardless the dose (5 tumours out of 260) a statistically significant difference ($P<0.05$ by χ-square test) was achieved *vs.* control mice (10 out of 171) and *vs.* 5-FU treated mice (9 out of 94).

Table IV summarizes FAA levels in plasma and tissues at different intervals after an i.v. dose of 150 mg kg$^{-1}$. Drug levels were highest in liver and kidney, lower and similar in tumour, colon and spleen. Though obviously three points are not sufficient for an accurate estimate of pharmacokinetic parameters, FAA plasma half-life was ~90 min. The rates of disappearance from tissues and plasma were very similar.

**Table IV** Mean plasma (μg ml$^{-1}$ and tissue (μg g$^{-1}$) levels (±s.e.) of FAA after i.v. injection of 150 mg kg$^{-1}$ in CF1 mice

| | *30 min*[a] | *60 min* | *120 min* |
|---|---|---|---|
| Plasma | 201±29 | 140±32 | 57±15 |
| Liver | 174± 4 | 130±25 | 55±14 |
| Kidney | 193±12 | 159±14 | 75± 4 |
| Spleen | 83± 7 | 58± 7 | 18± 5 |
| Colon | 108±14 | 73± 9 | 23± 3 |
| Tumour | 133±25 | 45±23 | 19± 8 |

[a]Groups consisted of 4 mice at 30 and 60 min, and of 3 mice at 120 min.

### Discussion

In the present study FAA reduced the number and burden of primary colonic tumours induced by MAM in mice. In previous *in vivo* studies evaluating the toxicity of FAA, the maximum cumulative tolerated dose was around 600 mg kg$^{-1}$ for a treatment period up to 20 days (Corbett *et al.*, 1986). In this study, a higher total dose could be given to mice in a longer period: in fact FAA doses up to 900 mg kg$^{-1}$ (150 mg kg$^{-1}$ for 6 times, weekly) were very well tolerated by all animals.

In previous studies, the FAA doses that showed significant antitumour activity were very close to the toxic ones (Corbett *et al.*, 1986). In contrast, in the experimental model we used, the antitumour effects of 70, 100 and 150 $mg\,kg^{-1}$ repeated doses were similar. Previous studies, however, employed s.c. transplanted tumours, whereas we investigated FAA activity in chemically induced tumours which grow in the colonic mucosa. We wonder whether the high susceptibility of MAM-induced tumours even to doses much lower than the toxic ones, might depend on their localization. The pharmacokinetic outline obtained however, does not indicate a preferential drug distribution in the colon tumours, where the FAA concentration was similar to or lower than that in other, normal tissues. Moreover *in vitro* studies on colon carcinoma cell lines showed growth inhibition at FAA concentrations greater than 400 $\mu g\,ml^{-1}$ for at least 24 hours (Bibby *et al.*, 1987; Capolongo *et al.*, 1987) and the FAA levels achieved in these primary tumours of mice after a dose of 150 $mg\,kg^{-1}$ appear to be too low to explain the observed tumour inhibition based only on a direct FAA cytotoxic effect. This reasoning is even more convincing when it is considered that doses of 70 and 100 $mg\,kg^{-1}$ were also effective against these tumours and presumably resulted in even lower FAA tumour concentrations.

Since the antitumour effects of FAA may be mediated by natural killer (NK) lymphocytes (Wiltrout, 1987; Ching & Baguley, 1987) and very high NK activity has been described in the murine gastrointestinal tract (Tagliabue *et al.*, 1981), these reasons could explain the effects achieved by FAA against primary colon tumours. A site-dependent sensitivity of tumours to FAA effects has already been reported by Double *et al.* (1987) and Giavazzi *et al.* (1988). In the former paper, the murine colon MAC 15 tumour responded to FAA when growing s.c. and not when cells were injected i.v. or i.p. In the latter study, a human colon tumour xenograft, virtually insensitive when growing s.c., was extremely sensitive to FAA when growing in the liver after intrasplenic injection. No definitive explanations for these discrepancies have been reported by either authors.

In the present study, a lower number of large tumours was found in FAA treated mice than in untreated control mice. Even though this observation should be supported by more detailed studies, FAA seems more effective against large than against small neoplastic lesions. This is unusual for an anticancer agent, but could be in keeping with the recent proposed hypothesis that FAA acts much the same way as tumour necrosis factor (TNF) (Smith *et al.*, 1987) and, like TNF, may be more effective on more advanced tumours (Manda *et al.*, 1987).

In conclusion, our study provides evidence that FAA is certainly effective against primary murine colon tumours, confirming the results against murine transplantable tumours. Moreover, mechanisms of action other than a direct cytotoxicity seem to be involved in its activity on murine models. Until now, however, no responses have yet been seen in early clinical investigation and this observation might reflect some fundamental difference between the way FAA actos in man and mouse. A better knowledge on mechanism of action of FAA might be needed as a basis for appropriate clinical investigations.

This work was supported in part by research grants from the Consiglio Nazionale delle Ricerche, Rome (Contracts No. 86.00704.44 and No. 86.02608.44). Giovanna Damia is recipient of a fellowship from the Italian Association for Cancer Research. The authors thank Dr Mary K. Wolpert of the Developmental Therapeutics Program, Division of Cancer Treatment, NCI, for supplying the FAA and Mr Enea Gandola for his excellent technical assistance.

### References

BIBBY, M.C., DOUBLE, J.A., PHILLIPS, R.M. & LOADMAN, P.M. (1987). Factors involved in the anti-cancer activity of the investigational agents LM985 (flavone acetic acid ester) and LM975 (flavone acetic acid). *Br. J. Cancer*, **55**, 159.

CAPOLONGO, L.S., BALCONI, G., UBEZIO, P. & 5 others (1987). Antiproliferative properties of flavone acetic acid (NSC 347512) (LM 975), a new anticancer agent. *Eur. J. Cancer Clin. Oncol.*, **23**, 1529.

CHING, L.-M. & BAGULEY, B.C. (1987). Induction of natural killer cell activity by the antitumour compound flavone acetic acid (NSC 347512). *Eur. J. Cancer Clin. Oncol.*, **23**, 1047.

CORBETT, T.H., BISSERY, M.-C., WOZNIAK, A. & 5 others (1986). Activity of flavone acetic acid (NSC 347512) against solid tumors of mice. *Investigational New Drugs*, **4**, 207.

DOUBLE, J.A., BIBBY, M.C. & LOADMAN, P.M. (1986). Pharmacokinetics and anti-tumour activity of LM985 in mice bearing transplantable adenocarcinomas of the colon. *Br. J. Cancer*, **54**, 595.

DOUBLE, J.A., BIBBY, M.C. & PHILLIPS, R.M. (1987). Site dependent sensitivity of a transplantable mouse colon tumour to flavone acetic acid (LM975). *Br. J. Cancer*, **56**, 215.

GIAVAZZI, R., GAROFALO, A., DAMIA, G., GARATTINI, S. & D'INCALCI, M. (1988). Response to flavone acetic acid (NSC 347512) of primary and metastatic human colorectal carcinoma xenografts. *Br. J. Cancer*, **57**, 277.

MANDA, T., SHIMOMURA, K., MUKUMOTO, S. & 8 others (1987). Recombinant human tumor necrosis factor-α: Evidence of an indirect mode of antitumor activity. *Cancer Res.*, **47**, 3707.

O'DWYER, P.J., SHOEMAKER, D., ZAHARKO, D.S. & 8 others (1987). Flavone acetic acid (LM975, NSC 347512). A novel antitumor agent. *Cancer Chemother. Pharmacol.*, **19**, 6.

PLOWMAN, J., NARAYANAN, V.L., DYKES, D. & 4 others (1986). Flavone acetic acid: A novel agent with preclinical antitumor activity against colon adenocarcinoma 38 in mice. *Cancer Treat. Rep.*, **70**, 631.

PRATESI, G. & DESCHNER, E.E. (1984). The antitumoral activity of 4′-deoxydoxorubicin compared to doxorubicin and 5-fluorouracil on methylazoxymethanol acetate-induced colon tumors in $CF_1$ mice. *Cancer*, **54**, 18.

PRATESI, G., GIANNI, L., MANZOTTI, C. & ZUNINO, F. (1988). Sequence dependence of the antitumor and toxic effects of 5-fluorouracil and cis-diamminedichloroplatinum combination on primary colon tumor in mice. *Cancer Chemother. Pharmacol.*, **21**, 237.

SMITH, G.P., CALVELEY, S.B., SMITH, M.J. & BAGULEY, B.C. (1987). Flavone acetic acid (NSC 347512) induces haemorrhagic necrosis of mouse colon 26 and 38 tumours. *Eur. J. Cancer Clin. Oncol.*, **23**, 1209.

TAGLIABUE, A., LUINI, W., SOLDATESCHI, D. & BORASCHI, D. (1981). Natural killer activity of gut mucosal lymphoid cells in mice. *Eur. J. Immunol.*, **11**, 919.

WILTROUT, R.H. (1987). Systemic augmentation of natural killer (NK) activity by the chemotherapeutic drug flavone-8-acetic acid. *Proceedings of the American Association for Cancer Research*, **28**, 347.